# First Record of Mating Involving a Melanistic Jaguar ( *Panthera onca* ) in the Wild: Novel Behavioural Insights Into Colour Morphs and Captive‐Wild Comparisons

**DOI:** 10.1002/ece3.71776

**Published:** 2025-08-25

**Authors:** Thomas Luypaert, Joseph E. Hawes, Raffaello Di Ponzio, Carlos A. Peres, Torbjørn Haugaasen

**Affiliations:** ^1^ Faculty of Environmental Sciences and Natural Resource Management Norwegian University of Life Sciences Ås Norway; ^2^ Institute of Science and Environment University of Cumbria Ambleside Cumbria UK; ^3^ Instituto Juruá Manaus AM Brazil; ^4^ Programa de Pós Graduação Em Ecologia, Conservação e Manejo da Vida Silvestre Universidade Federal de Minas Gerais Belo Horizonte Brazil; ^5^ School of Environmental Sciences University of East Anglia Norwich UK

**Keywords:** big cats, camera trapping, colour polymorphism, ex situ conservation, natural history, reproductive behaviour

## Abstract

Many threatened felid species, including the jaguar ( *Panthera onca* ), have low reproductive success in captivity. This may be partially attributed to a lack of knowledge on natural history parameters like courtship and mating behaviour in wild animal populations ‐ an essential aspect for fine‐tuning ex situ breeding programs. During a series of basin‐wide biodiversity inventories in the Brazilian Amazon, we captured videographic evidence of a mating event involving a melanistic jaguar. Such videographic evidence of natural jaguar mating behaviour remains rare, with prior behavioural descriptions being derived from artificial settings like zoos and wildlife parks. Our findings provide the first insights into the courtship and mating interactions between different jaguar colour morphs in the wild, and offer critical validation for behavioural data obtained from controlled environments. These insights enhance our understanding of jaguar reproductive biology and contribute valuable context for refining ex situ conservation strategies. More broadly, they highlight the role of natural history research in advancing conservation efforts for elusive species.

## Graphical abstract

We report the first scientifically documented copulation event between a melanistic and wild‐type jaguar ( *Panthera onca* ) in the wild, captured via remote camera in the Brazilian Amazon. The observed behaviours closely mirror those seen in captive jaguars, providing preliminary insights into the effects of melanism and captivity on jaguar mating interactions. These findings provide rare natural history insights that can inform and enhance ex situ conservation strategies for this threatened species.

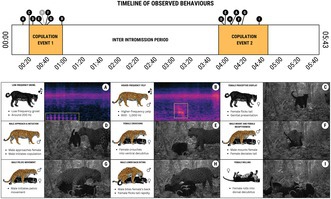

## 1 Introduction

The jaguar ( *Panthera onca* ) is the largest felid species in the Neotropics and the only extant member of the genus *Panthera* in the Americas (Quigley et al. 2017). As keystone predators, jaguars play a vital role in structuring food web dynamics and supporting ecosystem functioning across a range of habitats (Moreno et al. 2006; Ripple et al. 2014; Eriksson et al. 2022; Schmitz and Rizzuto 2024). Although being historically widespread, jaguar populations have suffered dramatic range contractions over the past 500 years due to habitat loss, hunting, and fragmentation (Morrison et al. 2007; Wolf and Ripple 2017). The Amazon remains their largest stronghold (Bogoni et al. 2023), yet many populations continue to decline, prompting their classification as ‘Near Threatened’ by the IUCN (IUCN 2024). To ensure long‐term survival of the species, one critical component of conservation efforts is the refinement of captive breeding programs and assisted reproductive technologies in ex situ settings, such as zoos and wildlife parks (Barnes et al. 2016; Jewgenow et al. 2017). These efforts are important for maintaining genetic diversity in populations facing imminent extinction in the wild and can enable future reintroduction initiatives (Wildt et al. 2010). For instance, such programs are vital for the Caatinga dry forest jaguar population, which is nearing extinction (Requena et al. 2023). However, many felid species, including jaguars, exhibit low reproductive success in captivity (e.g., Paz et al. 2000; Modena et al. 2023).

Compared to other felids, jaguar reproductive biology remains poorly understood (Ortiz et al. 2022). Existing knowledge is largely derived from studies conducted in captivity (e.g., Barnes et al. 2016; Andrews et al. 2019; Ortiz et al. 2022), which have advanced our understanding of physiological variables including hormone balances and oestrus cycles (Wildt et al. 1979; Barnes et al. 2016; Gonzalez et al. 2017), and led to the refinement of assisted reproductive techniques (Deco‐Souza et al. 2024), among others. Yet, natural mating behaviours ‐ including courtship, copulation and associated behavioural cues ‐ also influence reproductive success (Martin‐Wintle et al. 2017). Captivity has been shown to alter these behaviours in some Carnivora. For example, captive giant pandas ( *Ailuropoda melanoleuca* ) display atypical mating patterns and diminished sexual instincts relative to their wild counterparts, possibly due to psychological stress or mate incompatibility (Wang et al. 2024). In captive black‐footed ferrets ( *Mustela nigripes* ), approximately 25% of mating attempts failed because males did not adopt the appropriate copulatory posture (Wolf et al. 2000). In felids, captive breeding challenges may include the absence of visible oestrus, sexual incompatibility, or lack of interest in a selected mate (Wildt and Roth 1997). Crucially, copulatory cues such as vocalisations, mounting behaviour and postures, are closely tied to hormonal status, ovulation timing and mate receptivity, making them essential for successful breeding outcomes.

Captive studies have provided key insights into the mating behaviour of large felids, including jaguars. Stehlik (1971) offered a detailed account of a breeding event between jaguars at Ostrava Zoo (Czechia), describing oestrus signs, mating frequency and post‐copulatory behaviours. Building on such descriptive reports, Lanier and Dewsbury (1976) developed a behavioural classification system based on observations of four *Panthera* species ( *P. pardus* , *P. uncia* , *P. tigris* and *P. onca* ), including a spotted and melanistic jaguar pair. They identified six distinct behavioural patterns during mounts with intromission, as well as several latency measures related to phase durations and transitions (Lanier and Dewsbury 1976). More recently, Jorge‐Neto et al. (2018) extended this framework to 12 behavioural phases using year‐long videographic monitoring of a captive spotted jaguar pair in Brazil. Although these studies have expanded our understanding of jaguar mating behaviour, the importance of wild behavioural baselines remains underemphasized. As Wildt and Roth (1997) noted: ‘*studies on the reproduction of free‐ranging felids in range countries warrant attention, as these data are crucial for determining the norms for conspecifics in captivity*’. Yet, nearly three decades later, such data remain exceedingly rare.

The elusive and solitary nature of jaguars (Hunter 2018), combined with their extensive home ranges (Morato et al. 2016) and limited male–female interactions during brief oestrus periods (Ortiz et al. 2022), complicates the documentation of mating behaviours in their natural habitats. Currently, field observations of jaguar courtship and mating behaviour are limited. Leuchtenberger et al. (2009) reported a courtship attempt in the Pantanal involving scent marking and an unsuccessful mating approach towards a non‐receptive female. More recently, Fragoso et al. (2023) analysed 7 years of camera trap data from the Pantanal, recording 493 male–female interactions, including 381 consorts and 108 confirmed mating events. Despite deploying up to 77 camera traps annually, only four copulation events were captured on video ‐ highlighting the challenges of observing these behaviours in the wild. Importantly, both studies are geographically restricted to the Pantanal, and little is known about mating behaviours across the broader range of the species, which extends from southern Arizona (USA) to northern Argentina.

Beyond the influence of captivity on reproductive behaviour, another important yet underexplored aspect of jaguar biology is phenotypic variation in coat coloration, which may shape ecological adaptation, intraspecific niche partitioning and potentially mating behaviours (Heuer et al. 2024). Although the typical jaguar exhibits a spotted golden coat, a subset of the population displays melanism ‐ a condition caused by a dominant gain‐of‐function mutation in the MC1R gene that leads to increased eumelanin production and a predominantly black coat (Dittrich 1979; Eizirik et al. 2003). Melanism is relatively common among felids, occurring in 15 of 41 species (including the domestic cat), and has independently evolved at least eight times (Eizirik et al. 2003; Schneider et al. 2015; Kitchener et al. 2017; Graipel et al. 2019). In jaguars, approximately 10% of individuals are melanistic (da Silva 2017), though their distribution is nonrandom. Melanistic individuals are more frequently observed in the Amazon, Atlantic Forest and Cerrado, and are largely absent from the Llanos and Pantanal (da Silva 2017). This distribution suggests adaptive significance, consistent with Gloger's rule, which predicts darker pigmentation in humid climates due to camouflage and thermoregulatory benefits (Graipel et al. 2014; Delhey 2017). Notably, melanistic jaguars have been reported to exhibit greater daytime activity, which may enhance hunting success in brighter environments by improving camouflage (Mooring et al. 2020). Together, these findings indicate that melanism may provide fitness benefits and support niche differentiation between colour morphs (Mooring et al. 2020).

In addition to ecological implications, melanism may influence social and reproductive behaviours through pleiotropic effects, where a single genetic mutation affects multiple traits (Roulin and Ducrest 2011). Mutations within the melanocortin pathway ‐ including those associated with melanism ‐ have been linked to changes in sexual receptivity, fertility and aggression across taxa (Ducrest et al. 2008). Indirect effects may also arise if melanism alters intraspecific social interactions in species where colouration plays a role in mate recognition or cohesion (Caro et al. 2017). For example, deviant coat patterns in zebras ( *Equus quagga* ) are associated with reduced social bonding (Kingdon 1984), whereas in flycatchers ( *Monarcha castaneiventris* ), colour morph divergence has been implicated in reproductive isolation and incipient speciation (Uy et al. 2009). In jaguars, white ear markings, which enhance visibility in low‐light environments, are believed to play a key role in intraspecific communication (Ortolani 1999; Galván 2020). Their absence in melanistic individuals may hinder visual signalling, potentially offsetting camouflage advantages and helping maintain the evolutionary stability of both colour morphs (Graipel et al. 2019). These observations raise an important but unexplored question: do melanistic jaguars exhibit distinct mating behaviours that could influence reproductive dynamics in wild or captive populations?

To date, no study has explicitly investigated the role of captivity or melanism on jaguar courtship or copulation. Here, we present the first scientifically verified observation of a mating event involving a melanistic jaguar in its natural habitat, recorded via remote wildlife cameras (Video 1). We analyse the behavioural sequence in detail and compare it with published descriptions of mating behaviour in captive jaguars. These observations offer new insights into the reproductive ecology of jaguars and the potential role for captivity or melanism in shaping courtship dynamics. Beyond its novelty, this case study exemplifies the potential of natural history approaches (Tosa et al. 2021) to illuminate key gaps in the behavioural ecology of elusive species. Such insights are vital for informing conservation strategies, particularly for taxa that struggle to reproduce in captivity.

**VIDEO 1:** Videographic footage of the observed mating event between a melanistic and a wild‐type jaguar at Serra do Pardo National Park. The mating event consists of two consecutive mating sequences spanning approximately 6 min, and was captured on the 6th of September 2023 at around 14:21 Brasilia Time. Video content can be viewed at https://onlinelibrary.wiley.com/doi/10.1002/ece3.71776.

## 2 Methods

### 2.1 Data Collection

**FIGURE 1:**
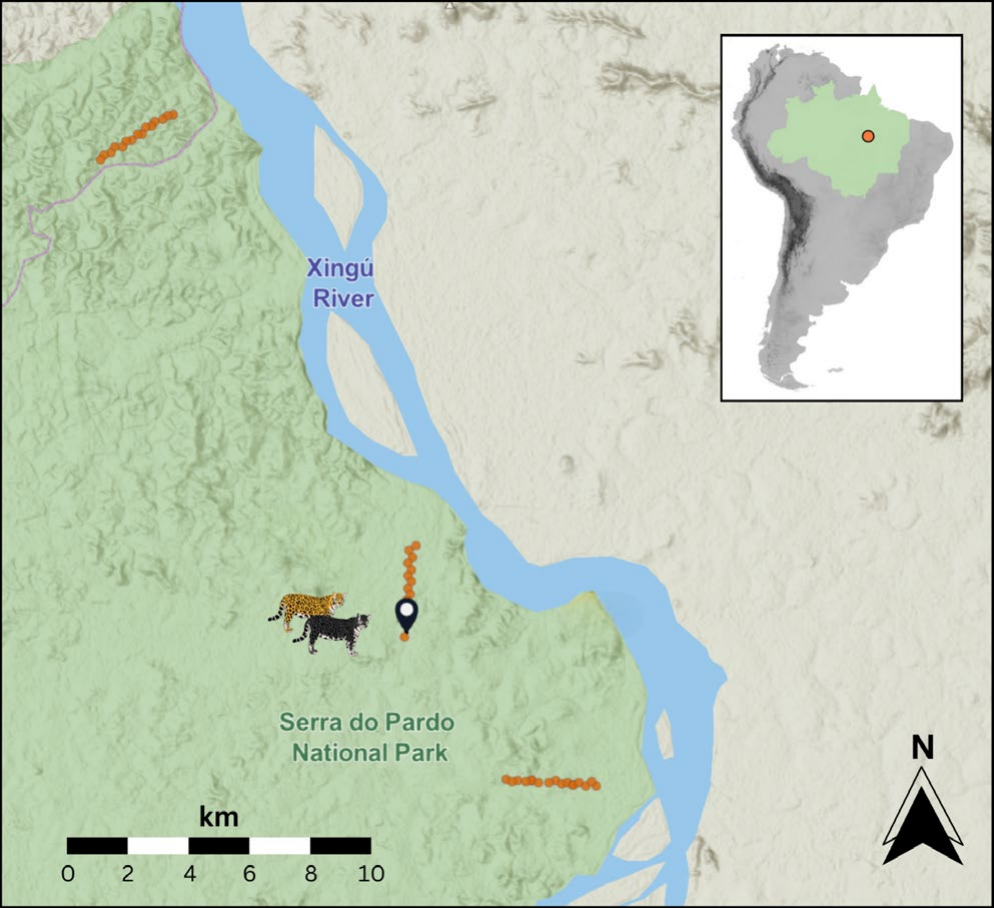
Map of the study area. The inset shows the sampling location (orange), in the Brazilian Amazon (green). The main map details Serra do Pardo National Park (green), where the study was conducted. Sampling transects are depicted with camera trap locations (orange). The location at which the jaguar pair was detected on the 6th of September 2023 between 14 h21 and 14 h29 BRT (Brasilia Time) is marked in black (5°48′29.3″ S–52°3827′01.9″ W). Map created using ESRI ArcGis online.

As a part of the Amazon Biodiversity and Carbon Expeditions (ABC Expeditions; www.abc‐expeditions.com), a rapid camera trap survey aimed at detecting large terrestrial vertebrates was conducted in Serra do Pardo National Park in the Brazilian Amazon between 6 and 20 September 2023. Three linear transects of 3‐km length were established on the western bank of the Xingú river, with each transect running perpendicular to the river bank, and the inter‐transect distance measured from the central transect being 9.6 and 16.2 km, respectively. A total of 48 camera traps (Browning Dark Ops; 16 per transect) were deployed simultaneously at 200 m intervals and at a 50 m perpendicular distance into the forest on alternate sides of the transect line (Figure 1). At each ‘off‐trail’ camera trap station, a single camera trap was mounted on a medium‐sized tree (20–50 cm diameter at breast height) at a height above ground of 30–50 cm. The cameras were unbaited and oriented northward towards sparsely vegetated areas approximately 2 m away, with vegetation cleared as needed to ensure unobstructed captures. Each camera was configured to record 10‐s videos per trigger, with a minimal delay of 1 s between triggers. Cameras remained in the field for 11 days, totalling 528 camera‐days across the three transects.

### 2.2 Behavioural Analysis of the Mating Event

The recorded jaguar detection event consisted of two distinct mating sequences, each of which was captured on camera. Following the approach described by Lanier and Dewsbury (1976) and Jorge‐Neto et al. (2018), we analysed each sequence individually, quantifying the presence and duration of various behavioural stages (Table 1). We defined the start and end of each mating sequence based on first and last observed behaviours in the known sequence of mating behaviours. In addition to the behavioural stages, we recorded contextual parameters, including the interval duration between consecutive mating sequences, time of year, and time of day. When a behavioural stage extended across multiple camera trap videos (each 10‐s in duration), we added 1 s to account for the trigger delay between consecutive recordings. Since the jaguars remained visible and active in front of the camera throughout the entire mating event, we assumed that the videos were captured continuously without any gaps in the recording interval. Finally, we determined the presence, duration, and core frequency of vocalisations by the heterosexual pair. To do so, we converted the mating event video from ‘.*mp4*’ to ‘.*wav*’ format for spectrogram visualisation in Audacity (version 3.7.0) and manually listened and inspected the audio sequence for jaguar sounds. Identified sounds were then overlaid with video footage to extract information on the mating sequence phases in which vocalisations occurred.

**TABLE 1:** Known behavioural phases during jaguar mating, modified from Lanier and Dewsbury (1976) and Jorge‐Neto et al. (2018).

Phase	Behavioural indicators
Female proceptive display	Female requests the male using proceptive display, including tail flicking and presentation of the anal‐genital region
Female vocalisation #1	Female vocalisation during proceptiveness
Male attraction	Male approaches the female and initiates copulation
Female crouching	Female indicates sexual receptiveness by crouching into ventral decubitus
Male mount	Male mounts the female from the back, keeping the female between his front paws while squatting to lower his genital region to the female's
Female receptiveness	Female accepts the male mount by deviating the tail to the side
Male pelvic movement	Male initiates pelvic movement, with or without penile penetration
Female vocalisation #2	Female vocalisation during penile penetration
Male nape‐biting/licking	Male licks or bites the female's nape during copulation
Male vocalisation	Male vocalisation during copulation, indicative of ejaculation
Female rocking	Female terminates copulation by striking the male
Female rolling	Female rolls into a dorsal decubitus after copulation

### 2.3 Signs of Lactation or Recent Parturition

In addition to the behavioural analysis, we assessed the videographic footage for signs of lactation or recent parturition, such as enlarged or swollen mammary glands/nipples, following Stasiukynas et al. (2022).

## 3 Results

The jaguar detection event reported here occurred at camera trap #15 (5°48′29.3″ S–52°38′01.9″ W) on the central monitoring transect (Figure 1) on the 6th of September 2023 between 14 h21 and 14 h29 BRT (Brasilia Time), which corresponds to nearly the midpoint of the two driest months of the year in a highly seasonal portion of the Amazon.

**FIGURE 2:**
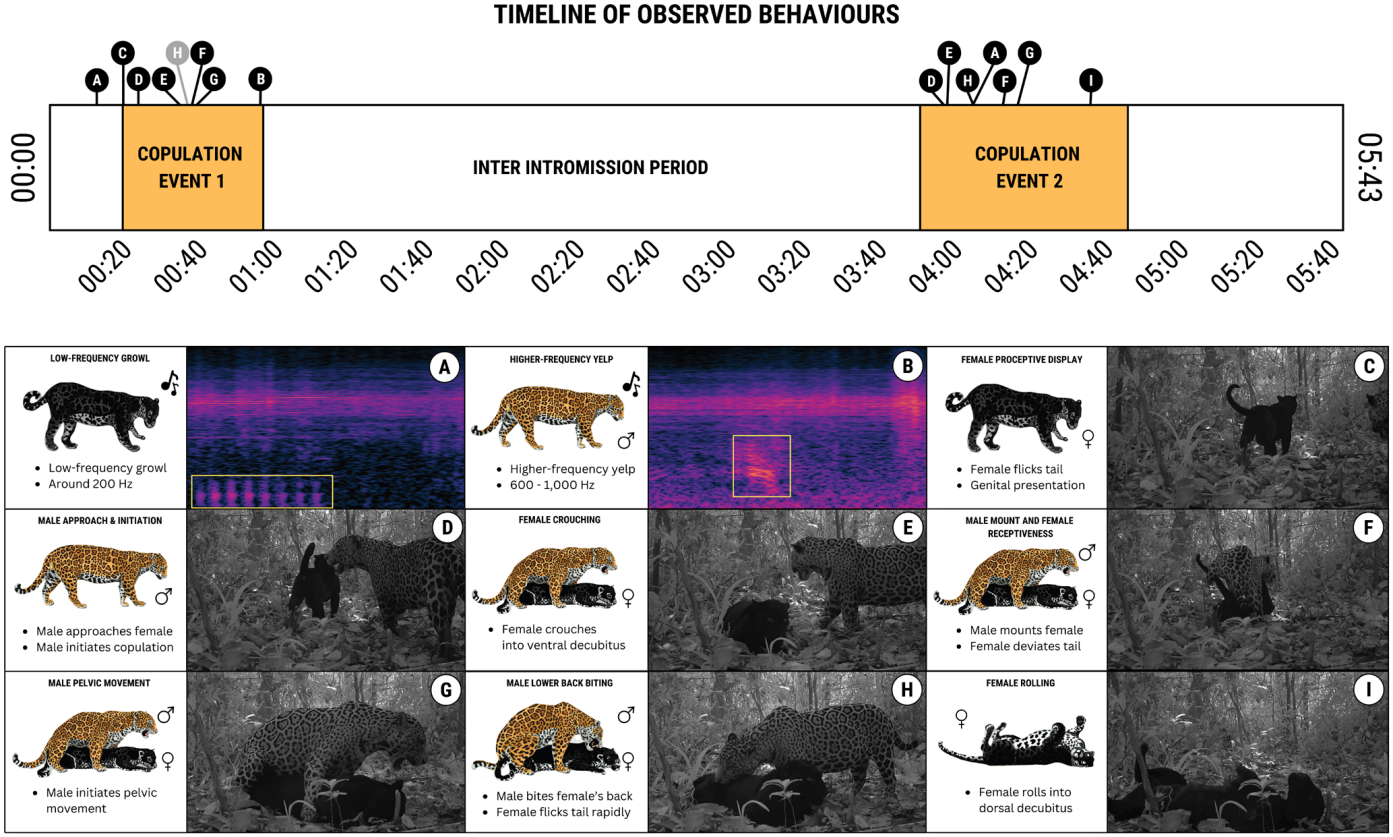
A chronological timeline of the jaguar mating event detected on the 6th of September 2023 between 14 h21 and 14 h29 BRT. The timeline is marked with the start of observed behavioural phases described in the legend below. The mating sequences are marked in orange. Behaviours that were always observed with certainty are marked in black, whereas behaviours that could not always be confirmed with certainty are marked in grey. Illustrations of jaguar mating phases by Pedro Busana are adapted from Jorge‐Neto et al. (2018) under the terms of the Creative Commons Attribution 4.0 International Licence.

We captured 35 10‐s videos of the jaguar pair, totalling 6 min and 17 s in front of the camera (Video 1). During this period, the jaguar pair copulated twice, and the associated behavioural phases (Table 1) and jaguar vocalisations are described in detail below in chronological order of occurrence. Across the two observed mating sequences, we recorded at least 9 out of the 12 commonly reported behavioural phases (Table 1; Figure 2). We provide the behavioural phases of the entire copulation event in a full‐length video (Video 1) as well as the corresponding timestamps in parentheses, matching the time of occurrence in the video (Figure 2).

Prior to the start of the first mating sequence, a series of low‐frequency growling noises was observed when the jaguar pair first entered the frame (timestamp: 00:13; frequency: 200 Hz). It was not possible to ascertain whether these vocalisations were emitted by the male or the female.

### 3.1 Mating Sequence #1 (00:20–00:57)

The first mating sequence was initiated by female proceptiveness displays, which were marked by tail‐flicking (00:20–00:35; duration: 16 s). During the proceptiveness phase, the male started to approach the female (00:24–00:38; 16 s). This was followed by the female crouching into ventral decubitus (00:35). At the end of the male approach, the male appeared to bite the female's lower back while she was already in ventral decubitus (00:37). However, this event was cut off by the end of the video. The male mounted the female from behind and the female was receptive, indicated by her deviating the tail to the side (00:38). The male initiated pelvic movement (00:40–00:57; 19 s). During copulation, the male appeared to undertake several attempts at biting the female's nape (00:45; 00:49; 00:53). However, whether a full nape‐bite occurred could not be established. At the end of pelvic movement, the male emitted a higher‐frequency yelp, upon which the male instantly dismounted the female (00:57; 600–1000 Hz). The male vocalisation and dismount marked the end of the first mating sequence, as the female did not perform rocking‐rolling behaviour following copulation. We also did not observe female vocalisation during proceptiveness or penile penetration in this mating sequence. In total, the first mating sequence lasted approximately 41 s.

Following the first mating sequence, the female remained in ventral decubitus and engaged in tail flicking and washing behaviours. During this period, the male remained nearby, first standing by the female (01:00–02:13), and later crouching into ventral decubitus (02:13–03:56). The interval between consecutive mating sequences lasted approximately 3 min and 17 s.

### 3.2 Mating Sequence #2 (03:51–04:46)

The second mating sequence was initiated when the female got up from ventral decubitus (03:51), which led to male attraction and approach (03:57), and female crouching into ventral decubitus again (03:58). While the female was in ventral decubitus, the male engaged in biting behaviour on the lower back of the female (04:05–04:12; 8 s), which led the female to flick her tail vigorously (04:09–04:12; 3 s). This behaviour was accompanied by a series of low‐frequency growling noises, presumably coming from the male (±200 Hz). Next, a second male mount was accepted by the female (04:13–04:16; 3 s). This was followed by male pelvic movement (04:17–04:27; 11 s), after which the male dismounted the female off‐camera. Whether the male dismount was preceded by nape‐biting or vocalisations could thus not be established. Following copulation, the female engaged in rolling behaviours, falling into dorsal decubitus and rolling on the forest floor (04:36–04:46; 11 s). This marked the end of the second mating sequence. During this mating sequence, we did not observe female proceptiveness displays, female vocalisation during penile penetration, or female rocking behaviours. In total, the second mating sequence lasted approximately 1 min and 1 s.

Following the second mating sequence, the female remained in ventral decubitus (04:46–05:40), with the male presumably standing nearby (not in frame). Finally, the female got up and left the frame (05:40) and was followed by the male. This was accompanied by a final acoustic event, constituting a series of low growling noises (200 Hz), presumably coming from the male. This marked the end of the jaguar detection event.

### 3.3 Signs of Lactation or Recent Parturition

The female showed potential signs of lactation, including nipple development which can be observed during the rolling phase (04:36–04:46; Figure 3).

**FIGURE 3:**
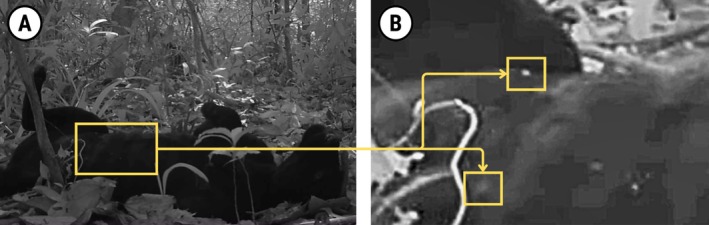
Potential indicator of lactation or recent parturition in a female jaguar, showing visibly swollen nipples during the rolling behaviour phase (A) and in close‐up detail (B). These features correspond to Video 1 at approximately 04:38.

## 4 Discussion

This study presents the first scientifically documented videographic footage and detailed description of copulatory behaviour involving a melanistic jaguar in the wild. To our knowledge, it also constitutes the only direct observation that allows for comparison between wild and captive jaguar mating behaviour. Although this single observation does not permit broad conclusions, the sequence of observed behavioural phases aligns closely with those previously reported in captive individuals, capturing 9 out of 12 known behavioural phases. This suggests a possible degree of behavioural consistency across both environments and colour morphs. We emphasise that further observations are necessary to determine whether this similarity reflects true behavioural conservation or individual variation.

### 4.1 Behavioural Phases

Compared to previously described behavioural phases during the jaguar mating sequence in captivity (Stehlik 1971; Lanier and Dewsbury 1976; Jorge‐Neto et al. 2018), there were minimal differences in the observed mating behaviours for the jaguar pair in the wild observed here. Both sequences included male approach, female crouching, male mounting, female receptiveness, and male pelvic movement. However, some behaviours were only observed in one of the sequences. For example, female proceptive displays and male vocalisations during copulation were present only in the first event (although male vocalisations could not be established in the second event due to the video ending). Female rolling behaviours were only seen in the second mating event. Although there were indications that the male attempted nape biting or licking, we could not confirm these actions. Additionally, some behaviours did not fully align with previously described behavioural phases. For instance, we also observed the male biting the female's lower back while growling during the second mating sequence, and potentially in the first as well. This action caused the female to flick her tail vigorously. Although the exact significance of this behaviour remains unclear, it may be part of the pre‐copulatory courtship, possibly helping to stimulate the female's sexual receptivity.

We did not detect female vocalisations during proceptiveness or penile penetration. Given the poor audio quality and background noise, it was not possible to confirm whether these vocalisations occurred. Similarly, female rocking behaviour, in which the female strikes the male after copulation, was not observed. This absence aligns with findings from Jorge‐Neto et al. (2018), who observed female rocking in only 58% of copulations with penile penetration, and with Lanier and Dewsbury (1976), who noted this behaviour in other felid species but not in jaguars. Finally, male post‐copulatory behaviours described by Stehlik (1971), such as drinking, urination, and genital licking, were not observed. Although the lack of drinking can easily be explained by the absence of a water source, urination or genital grooming may have occurred off‐camera, as the male briefly left the frame between the two mating events and after the second event.

In general, the mating behaviours we observed were consistent with those reported in captive jaguars. Although colour patterns are known to play a role in visual communication among jaguars (Graipel et al. 2019), our single observation did not reveal any overt behavioural differences associated with melanism. Auditory and olfactory cues also influence feline mating behaviour (Jorge‐Neto et al. 2020), and if these stimuli are the primary drivers of mating behaviours, this could explain the lack of behavioural differences related to melanistic colouration. Although melanism‐induced behavioural changes are sometimes linked to pleiotropic effects, these genetically induced changes are generally associated with the regulation of melanocortin peptides (Ducrest et al. 2008). Mutations in genes like MC1R, responsible for melanism in jaguars, are believed to have minimal pleiotropic effects (Mundy 2005), which aligns with our observed absence of behavioural changes. This genetic context may help explain the absence of obvious behavioural divergence in our case.

However, given the limitations associated with an opportunistic single observation, we cannot conclusively determine whether melanism has any influence, direct or indirect, on mating behaviour. The lack of certain behaviours in our recordings may also reflect the constraints of camera trap monitoring, rather than true differences in natural behaviour. Our findings offer a rare glimpse into melanistic jaguar mating behaviour in the wild, but further data are needed to assess whether these patterns are consistent across individuals and contexts.

### 4.2 Intromission and Copulation Timing

Although we were unable to confirm whether penile intromission occurred from our camera trap data, several behavioural phases observed in this study align with successful intromission and ejaculation. For instance, the distinctive male vocalisation at the end of the first mating sequence matches the ‘scream’ observed by Lanier and Dewsbury (1976) in 100% of mating events with intromission. Similarly, Jorge‐Neto et al. (2018) recorded male vocalisation in all mating events with penile introduction, but only in one out of 88 events without intromission, suggesting that male vocalisation is a reliable indicator of successful ejaculation. Additionally, the female rolling behaviour observed after the second mating further supports the likelihood of successful intromission and ejaculation in at least one of the two observed mating events, as also noted by Stehlik (1971) and Jorge‐Neto et al. (2018).

Comparing the duration of copulation in our observations with previous reports is challenging due to the lack of precise timing for penile penetration and differing definitions of ‘copulation duration’ across studies (e.g., duration of intromission vs. overall mating sequence). Stehlik (1971) estimated the average duration of coitus in jaguars at 9 s (ranging from 2 to 35 s), with a pre‐intromission mount duration of 5–13 s. Our observed male pelvic movements (19 and 11 s in the two copulation events, respectively), which likely included both the mount and intromission phases, fall within this range. Similarly, Fragoso et al. (2023) reported copulation durations between 5 and 112 s. Although our measurements of male pelvic movement fall within this range, it is unclear which specific phases of mating were being measured in Fragoso et al. (2023). The same study also found that jaguar mating predominantly occurs in the early morning or after 16 h. In contrast, our observed mating occurred around 14 h30, likely reflecting regional differences in environmental conditions. In the Brazilian Pantanal, where Fragoso et al. (2023) made the observations, higher midday temperatures may influence the timing of mating events for jaguars.

These behavioural cues, such as the timing of pelvic thrusts, male vocalisation, and female rolling behaviour, could help refine assessments of mating success in both wild and captive settings. In ex situ breeding programs, such indicators may be valuable for evaluating mating compatibility or identifying the optimal timing for interventions such as artificial insemination.

### 4.3 Inter‐Intromission Duration and Mating Frequency

Jaguars often perform several copulations per day during female oestrus. Our study was unable to determine the frequency of copulation events for the wild jaguar pair, as our camera station recorded only two events before the pair moved out of frame. However, within a span of approximately 5 min, we captured two copulation events with an inter‐intromission interval of about 3 min.

This rapid succession of mating events aligns with observations in captive jaguars. According to Stehlik (1971), the frequency of mating depends on the female oestrus. These authors reported 10–20 mating events per day during peak oestrus, but only 2–3 mating events near the end of oestrus, with the mating frequency being much higher when captive jaguars were separated during most of oestrus. Lanier and Dewsbury (1976) reported the inter‐intromission interval to be between a few minutes and an hour, and Fragoso et al. (2023) reported a minimum inter‐intromission interval of 1 min. The short inter‐intromission interval observed in this study could indicate that the female was in peak oestrus when the mating events occurred (but see next section), and mating likely proceeded off‐camera. Furthermore, it seems that short inter‐intromission intervals occur naturally and are not the product of jaguar separation during oestrus, as posited by Stehlik (1971). This rapid succession of mating events is consistent with the hypothesis that frequent mating helps induce ovulation in female jaguars, as they are primarily induced ovulators.

### 4.4 Signs of Female Lactation and Potential Hide‐And‐Flirt Behaviour

A close‐up analysis of the female's abdominal region during the rolling phase revealed possible indicators of lactation or recent parturition, such as visibly swollen mammary glands and nipples (Figure 3). However, due to the grainy quality of the footage and the absence of comparative data, such as prior footage of the same individual before potential pregnancy or visual confirmation of her with dependent offspring, it is not possible to confirm lactation with certainty.

If this interpretation is correct, it may suggest that the female was not in true oestrus, as suggested before, but instead exhibiting pseudo‐oestrus: oestrus‐like behaviour not directly associated with immediate reproduction (Benson et al. 2012). Pseudo‐oestrus has been proposed as an adaptive strategy in which lactating females engage in mating behaviours with males while concealing their young, known as the ‘hide‐and‐flirt’ strategy. This behaviour may serve to confuse paternity or reinforce social bonds with males, thereby reducing the likelihood of infanticide (Benson et al. 2012). Another proposed function of non‐conceptive mating is to deplete the sperm reserves of males, thereby reducing fertilisation success in rival females and potentially decreasing future reproductive competition (Sommer et al. 1992; Doran‐Sheehy et al. 2009).

Evidence of non‐conceptive mating in jaguars has previously been reported in the Pantanal and Llanos regions, where Stasiukynas et al. (2022) documented three instances of females copulating with males while raising cubs. Similarly, Fragoso et al. (2023) reported observations consistent with the hide‐and‐flirt strategy in jaguars from the Pantanal.

## 5 Conclusion

Although understanding jaguar reproductive behaviour is critical for refining ex situ breeding programs and better understanding in situ conservation, direct evidence from natural mating events has been largely lacking in the literature. This study provides the first benchmark of mating behaviours in a wild jaguar pair, with an explicit comparison to captive jaguars. Although based on a single observation, our findings show broad similarities to previously documented copulatory behaviours in captivity. This preliminary result suggests that copulatory behaviour may be relatively consistent across environments, though further observations of jaguar mating in the wild are needed to confirm this.

This study also documents, for the first time, a mating event involving a melanistic jaguar in the wild. Although no distinct behavioural differences were observed, future research should investigate whether melanistic and non‐melanistic individuals differ in reproductive behaviour and success, mate selection, or other fitness‐related traits. Long‐term, systematic monitoring of mating events in both wild and captive settings ‐ ideally with known genetic identities and reproductive outcomes ‐ will be essential to determine whether colour morphs are associated with meaningful behavioural or fitness differences.

Beyond its descriptive value, our study contributes to a deeper understanding of jaguar reproductive strategies. For example, the potential expression of *‘*hide‐and‐flirt’ behaviour ‐ whereby a lactating female consorts with a male while concealing her cubs ‐ lends support to previous reports of pseudo‐oestrus in jaguars. Such behaviour may function to confuse paternity or reduce the risk of infanticide, highlighting the complex and potentially adaptive nature of jaguar mating systems. Observations like these underscore the importance of documenting natural behaviour in the wild to capture the full breadth of reproductive strategies in this species.

Our findings also have practical relevance for both in situ and ex situ conservation strategies. For captive breeding programs, the behavioural consistencies observed here may help inform pair compatibility assessments and optimise the timing of interventions such as artificial insemination. In wild populations, documenting natural mating behaviour provides important baseline information for understanding reproductive ecology, which can support efforts to monitor population health, reproductive output, and complex social dynamics under varying environmental conditions. Such data are also valuable for assessing how different colour morphs, such as melanistic individuals, participate in key reproductive behaviours in the wild.

Finally, this study highlights the critical role of natural history observations in remote tropical ecosystems. Opportunistic, high‐resolution behavioural data can provide critical insights on the life history of elusive species such as the jaguar ‐ directly informing conservation management, improving reproductive success in captivity, and guiding future ecological research.

## Author Contributions

**Thomas Luypaert:** conceptualization (lead), data curation (lead), formal analysis (lead), investigation (lead), methodology (lead), visualization (lead), writing – original draft (lead). **Joseph E. Hawes:** conceptualization (supporting), formal analysis (supporting), funding acquisition (supporting), investigation (supporting), methodology (supporting), project administration (supporting), supervision (supporting), writing – original draft (supporting). **Raffaello Di Ponzio:** conceptualization (supporting), formal analysis (supporting), investigation (supporting), methodology (supporting), visualization (supporting), writing – original draft (supporting). **Carlos A. Peres:** conceptualization (supporting), formal analysis (supporting), funding acquisition (lead), investigation (supporting), methodology (supporting), project administration (lead), supervision (lead), writing – original draft (supporting). **Torbjørn Haugaasen:** conceptualization (supporting), formal analysis (supporting), funding acquisition (lead), investigation (supporting), methodology (supporting), project administration (lead), supervision (lead), writing – original draft (supporting).

## Conflicts of Interest

The authors declare no conflicts of interest.

## Data Availability

The full‐length video documenting the observed jaguar copulation event is available as embedded rich media in the manuscript.
